# Simultaneous Electrochemical Detection of Catechol and Hydroquinone Based on a Carbon Nanotube Paste Electrode Modified with Electro-Reduced Graphene Oxide

**DOI:** 10.3390/ijms25189829

**Published:** 2024-09-11

**Authors:** Tingfei Chen, Chao Liu, Xiaojun Liu, Chunnan Zhu, Dongyun Zheng

**Affiliations:** 1 School of Biomedical Engineering, South-Central Minzu University, Wuhan 430074, China; chentingfei2622@163.com (T.C.); chaoliu@scuec.edu.cn (C.L.); toliuxiaojun@hotmail.com (X.L.); cnzhu@mail.scuec.edu.cn (C.Z.); 2 Key Laboratory of Cognitive Science, State Ethnic Affairs Commission, Wuhan 430074, China; 3 Hubei Key Laboratory of Medical Information Analysis and Tumor Diagnosis & Treatment, Wuhan 430074, China

**Keywords:** graphene, carbon nanotube, catechol, hydroquinone, modified electrode, simultaneous detection

## Abstract

Effectively detecting catechol (CC) and hydroquinone (HQ) simultaneously is crucial for environmental protection and human health monitoring. In the study presented herein, a novel electrochemical sensor for the sensitive simultaneous detection of CC and HQ was constructed based on an electrochemically reduced graphene oxide (ERGO)-modified multi-walled carbon nanotube paste electrode (MWCNTPE). Scanning electron microscopy, X-ray photoelectron spectroscopy, Raman spectroscopy and electrochemical techniques were utilized to characterize the sensing interface and investigate the sensing mechanism. Under the optimal detection conditions, the oxidation peak currents of CC and HQ show a good linear relationship with their concentrations in the range of 0.4–400 μM with a detection limit of 0.083 μM for CC and 0.028 μM for HQ (S/N = 3). Moreover, the sensor exhibits good performance and can be applied successfully in the simultaneous detection of CC and HQ in tap water samples and urine samples with satisfactory results, indicating its promising application prospects.

## 1. Introduction

Catechol (CC) and hydroquinone (HQ) are isomers of dihydroxybenzene, and are known as environmental endocrine disruptors [1]. As metabolites of benzene, phenols are considered important indicators for monitoring the metabolism of the carcinogen benzene in the human body [2]. It has been proven that they can affect the human endocrine system and are associated with endocrine tumors [3]. Excessive amounts of CC and HQ may also cause fatigue, tachycardia, liver damage, kidney dysfunction, and other health issues [4]. However, the direct and simultaneous determination of the isomers presents a challenge due to their similar properties [5]. Therefore, it is crucial to develop an efficient, sensitive, selective, and low-cost method for the detection of CC and HQ.

Various methods have been used for the simultaneous detection of CC and HQ, including spectrophotometry [6], fluorescence [7], chemiluminescence [8], high-performance liquid chromatography [9] and electrochemical analysis [10]. Among these methods, electrochemical analysis has been widely used for detection in a range of applications, from humans to the environment, due to its simple operation, low cost and fast response [11,12,13,14]. However, extended overlapping voltammetric signals may affect the transient detection of CC and HQ due to their similar properties [15]. Therefore, it is necessary to develop an effective electrochemical sensor to enhance selectivity and sensitivity for CC and HQ detection.

Nanomaterials such as metal nanoparticles [16] and carbon nanomaterials (i.e., carbon nanofibers [17], fullerenes [18], carbon nanotubes [19], and graphene [20]) have been widely used to modify electrode surfaces. Graphene (G) and multi-walled carbon nanotubes (MWCNTs) have become promising materials in the field of electrode modification due to their excellent properties and environmental friendliness [21,22,23,24]. However, G is extremely vulnerable to self-polymerization or stacking due to Van der Waals interactions, resulting in a loss of surface area and electrical conductivity [25]. Therefore, reduced graphene oxide (RGO) was synthesized by reducing graphene oxide (GO) to remove the oxygen groups. This process not only retains the high electrical conductivity and mechanical strength of graphene but also maintains the dispersion and functionalization of GO [26,27]. Several methods have been used to produce RGO, including chemical reduction [28], thermal reduction [29], and electrochemical reduction [30]. Compared to these methods, the electrochemical reduction of GO (ERGO) not only produces high-quality material but also forms ERGO films directly on the electrode surface through a one-step electrodeposition technique.

Glassy carbon electrodes (GCEs) are extensively utilized in developing CC and HQ electrochemical sensors because of their facile surface modification and reproducibility. However, these electrodes often face challenges such as low surface activity, poor adsorption of modifying materials, and high costs, that prevent them from being disposable [31,32]. Hence, there is a need to develop a cost-effective and easy-to-prepare disposable electrode. In recent years, carbon materials have gained extensive utilization in electrochemical research due to their excellent properties, eco-friendly nature, and cost-effectiveness [33,34,35]. Multi-walled carbon nanotubes (MWCNTs), consisting of multiple layers of graphite rolled into tube shapes with varying diameters, exhibit several remarkable properties, including a larger specific surface area, higher stability, and better electrical conductivity, even in smaller amounts compared to graphite [36,37]. When MWCNTs are used as an electron transfer medium, they significantly reduce the overpotential of the redox reactions of detected substances, thereby enhancing the electrode’s selectivity and sensitivity [38]. Therefore, MWCNTs are widely used as the primary prepared carbon material for carbon paste electrodes [11,39,40].

**Scheme 1:**
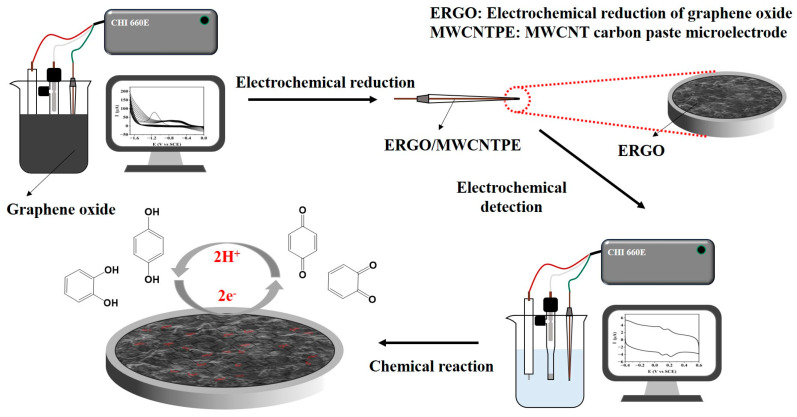
Schematic diagram showing the preparation of the ERGO/MWCNTPE and its application in the electrochemical detection of CC and HQ.

In this work, we developed a disposable ERGO-modified MWCNT carton paste microelectrode (ERGO/MWCNTPE) using a simple and controllable electrodeposition method for the simultaneous detection of CC and HQ. The sensitivity and selectivity of the sensor were enhanced due to the synergistic interaction of MWCNT and ERGO on the surface of the modified electrode. Scheme 1 describes the preparation of the ERGO/MWCNTPE and the electrochemical affect of CC and HQ on the electrode. The electrochemical sensor was characterized, and its performance in detecting CC and HQ was also investigated. The results showed that the ERGO/MWCNTPE exhibited good electrocatalytic performance for the redox reactions of CC and HQ. It was effective in the determination of CC and HQ in tap water samples and urine samples with satisfactory results.

## 2. Results and Discussion

### 2.1. Electrochemical Reduction of Graphene Oxide

**Figure 1:**
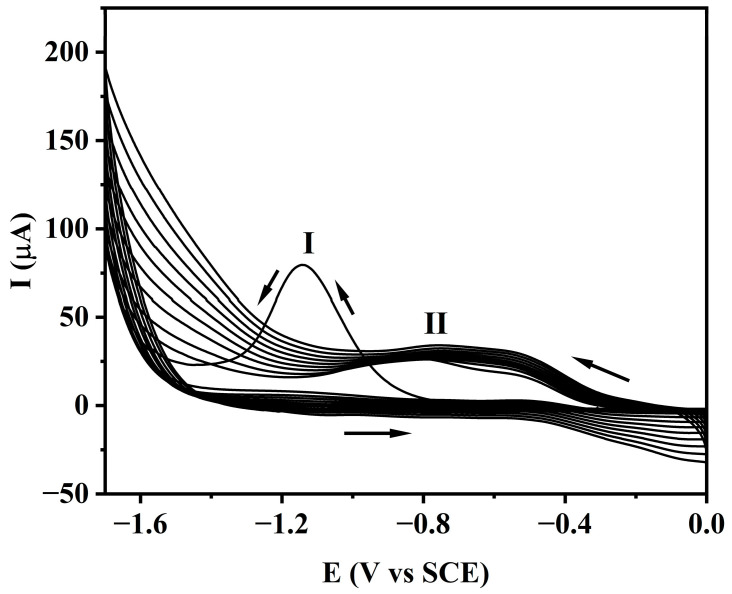
Cyclic voltammograms of the electrochemical reduction of GO on the MWCNTPE.

To improve the conductivity and electrocatalytic properties of the electrodes, graphene oxide was electrochemically reduced. As shown in Figure 1, ERGO was generated by reducing the oxygen groups on the GO film using cyclic voltammetry, which involved applying a high negative potential ranging from 0 V to −1.7 V. The cathodic peak I within the first cycle, which had the largest current signal at a potential of −1.1 V, suggests that the process of GO reduction was almost complete [25,41]. In the subsequent cycles, the remaining reactive oxygen groups on the GO surface were gradually reduced, corresponding to peak II. Only a small portion of the oxygen-containing groups was retained to improve the adsorption of CC and HQ.

### 2.2. Morphological and Structural Characterization of Different Electrodes

**Figure 2:**
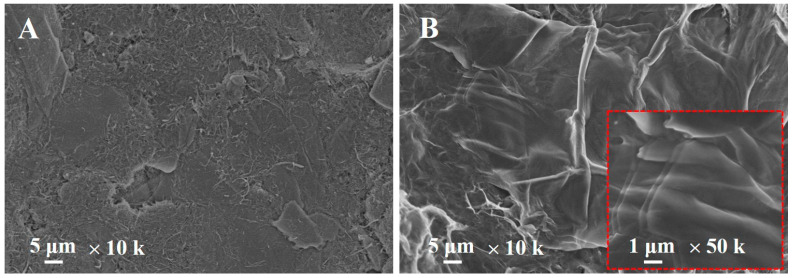
SEM images of the bare MWCNTPE (**A**) and ERGO/MWCNTPE (**B**).

The morphology of the different electrode surfaces was characterized via scanning electron microscopy (SEM). As shown in Figure 2A, numerous striped MWCNTs were observed on the electrode surface, indicating that the MWCNTs mixed with paraffin oil were compact. Figure 2B shows that the MWCNTs were completely obscured by a large number of bulging ERGO flakes, indicating that the effective area of the electrode was expanded, thereby improving the enrichment of HQ and CC.

**Figure 3:**
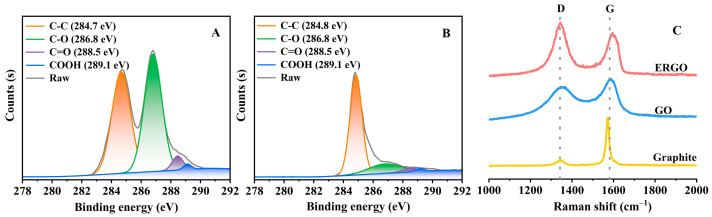
XPS spectra of GO (**A**)and ERGO (**B**) and Raman spectra (**C**) of graphite, GO and ERGO.

The structures of ERGO and GO were characterized using XPS and Raman spectroscopy. As shown in Figure 3A,B, the intensity of the C1s peaks corresponding to oxygen-bound carbon atoms in ERGO was significantly reduced compared to GO, indicating that the electrochemical reduction was relatively thorough and most of the oxygen-containing groups were removed. To further understand the electronic properties of graphite, GO, and ERGO, Raman spectra were analyzed (Figure 3C). The G peak corresponds to the in-plane vibration of the E_2_g symmetric mode of carbon–carbon bonding in graphite crystals, and its presence indicates higher crystal quality. The D peak originates from the vibration of the A_1_g symmetric mode due to lattice defects or the irregular arrangement of carbon atoms, with a stronger D peak indicating greater structural defects [42]. Therefore, the defects in graphite, GO, and ERGO increase sequentially, suggesting that the electrochemical reduction of GO further increases the structural defects resulting from graphite oxidation.

### 2.3. Electrochemical Behavior of the Modified Electrode

**Figure 4:**
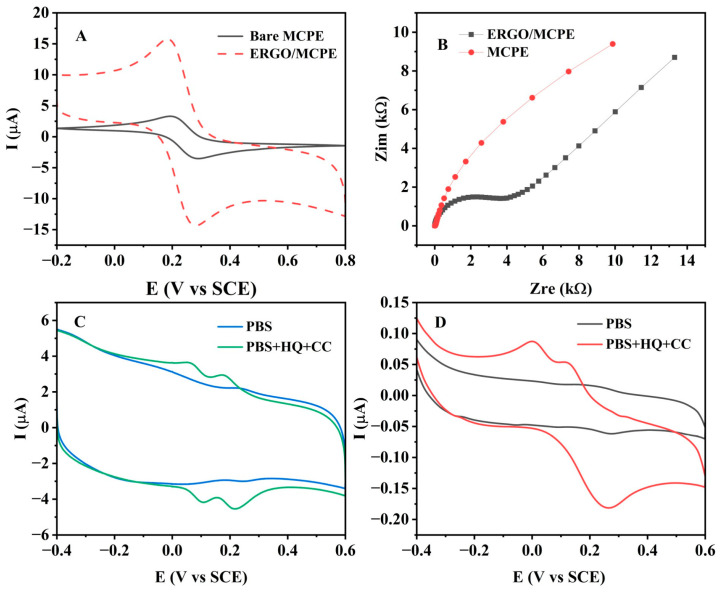
Cyclic voltammograms of the bare MWCNTPE and ERGO/MWCNTPE in 5.0 mM [Fe(CN)_6_]^3−/4−^ solution containing 0.1 M KCl (**A**). EIS of the bare MWCNTPE and ERGO/MWCNTPE in 5.0 mM [Fe(CN)_6_]^3−/4−^ solution containing 0.1 M KCl (**B**). Cyclic voltammograms of bare MWCNTPE (**D**) and ERGO/MWCNTPE (**C**) in 0.2 M PBS (pH = 6) with or without 0.1 mM CC and HQ.

Cyclic voltammetry (CV) is an electrochemical technique used to measure the current response of a redox-active solution to a linearly cycled potential sweep using a potentiostat. It is a useful method for quickly obtaining information about the kinetics of electron transfer reactions. Using [Fe(CN)_6_]^3−/4−^ as a probe, cyclic voltammetry was applied to investigate the electrochemical properties of different electrodes. As shown in Figure 4A, only a weak redox peak was observed on the bare MWCNTPE (solid line). However, following modification with ERGO, the redox current increased significantly (dashed line). This change is attributed to the excellent electrical conductivity, large active area, and residual oxygen functional groups of the ERGO films [43,44].

Electrochemical impedance spectroscopy (EIS) was further used to investigate the interface properties of the electrode surfaces. In a typical Nyquist plot, the EIS curve consists of two parts. One is a semicircle in the high-frequency region, which is usually controlled by diffusion. The other is a straight line in the low-frequency region, which is related to electron transfer resistance. The diameter of the semicircle is positively correlated with electron transfer resistance [45]. In Figure 4B, [Fe(CN)_6_]^3−/4−^ was used as a redox probe to study the surface characteristics of different electrodes. The semicircle diameter of the bare MWCNTPE is larger than that of the ERGO/MWCNTPE, which indicates that the electrical conductivity of the ERGO/MWCNTPE is better. This finding is attributed to the excellent electrical conductivity of both MWCNTs and ERGO. The results are consistent with the CV curves, which further proved that ERGO-MWCNTs effectively improve electrical conductivity and accelerate electron transfer.

The electrocatalytic performance of different electrodes was investigated by using cyclic voltammetry in 0.2 M PBS buffer solution (pH = 6) containing 0 mM or 0.1 mM CC and HQ. The results are shown in Figure 4C,D. When no CC and HQ were present in the electrolyte solution, almost no electrochemical response was observed. When 0.1 mM CC and HQ were present in the electrolyte solution, two distinct oxidation peaks for CC and HQ were observed on the ERGO/MWCNTPE: E_pa(CC)_ = 0.223 V, I_pa(CC)_ = 0.769 μA, E_pc(CC)_ = 0.179 V, and I_pc(CC)_ = 0.494 μA for CC, and E_pa(HQ)_ = 0.105 V, I_pa(HQ)_ = 0.508 μA, E_pc(HQ)_ = 0.059 V, and I_pc(HQ)_ = 0.418 μA for HQ. This finding indicates that a quasi-reversible reaction occurs on the surface of the ERGO/MWCNTPE. However, only one oxidation peak was observed on the bare MWCNTPE with the following parameters: E_pa_ = 0.265 V, I_pa_ = 0.099 μA, E_pc(HQ)_ = 0.000 V, I_pc(HQ)_ = 0.033 μA, E_pc(CC)_ = 0.127 V, and I_pc(CC)_ = 0.020 μA. These findings indicate that bare MWCNTCPE cannot separate the oxidation peaks of CC and HQ. In contrast, the ERGO/MWCNTPE demonstrates strong catalysis and high separation of the oxidation peak for CC and HQ. This is attributed to the oxygen-containing functional groups and defect structures of ERGO, as well as the high conductivity, electron transfer kinetics and catalytic activity of ERGO-MWCNTs.

### 2.4. Optimization of Experimental Conditions

The electrode optimization processes are detailed in the Supplementary Materials. The optimal conditions were as follows: (1) a 1 mg∙mL^−1^ of GO dispersion with 10 cycles for ERGO preparation (Figure S1A,B); (2) a 0.2 M PBS buffer solution with a pH of 6.0 as the supporting electrolyte (Figures S1E and S2); (3) −0.2 V for enrichment potential (Figure S1D) and 50 s for enrichment time (Figure S1C). The response of the sensor in this work is a little slow, which could be accelerated through using a smaller electrochemical cell in our future research.

### 2.5. Influence of the pH Value of the Electrolyte

**Figure 5:**
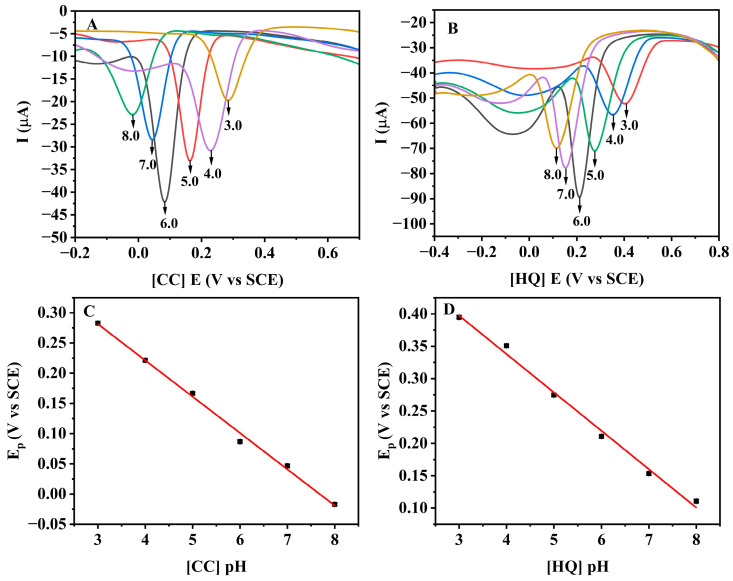
Square wave voltammograms of 0.1 mM CC (**A**) and HQ (**B**) in 0.2 M PBS on the ERGO/MWCNTPE at different pH values (from left to right: 3.0, 4.0, 5.0, 6.0, 7.0, and 8.0, respectively). The linear relationship between the oxidation peak potentials of CC (**C**) and HQ (**D**) and the pH of the electrolyte are shown.

Square wave voltammetry (SWV) is certainly one of the most advanced and versatile forms of pulse voltammetry. It offers high analytical sensitivity and speed of measurement [46]. The pH value of the electrolyte affects the electrostatic interaction between the sensing interface; CC or HQ was investigated via SWV (Figure 5A,B). Within the pH range of 3.0 to 8.0, the peak potential (E_pa_) of CC and HQ decreased linearly with increasing pH (E_pa, CC_ (V) = 0.575–0.059 pH, R^2^ = 0.99, E_pa, HQ_ (V) = 0.462–0.060 pH, and R^2^ = 0.99) (Figure 5C,D). This finding indicates that protons are involved in the electrochemical redox processes of CC and HQ at the sensing interface, and that the number of protons and transferred electrons involved in the electrochemical oxidation of CC and HQ are the same [47].

### 2.6. Influence of the Scan Rate

**Figure 6:**
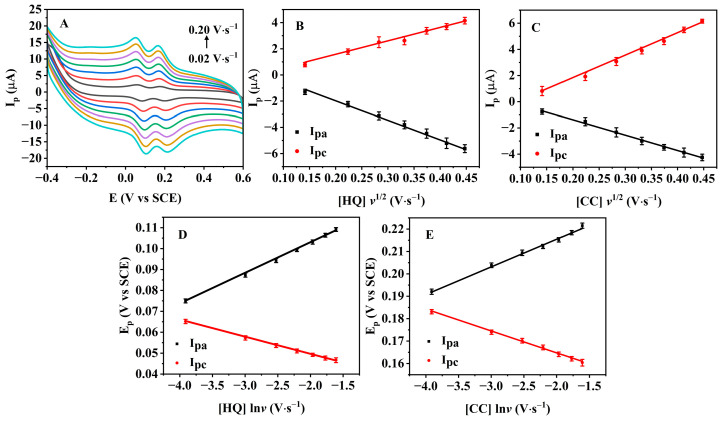
Cyclic voltammograms of the ERGO/MWCNTPE in 0.2 M PBS (pH = 6.0) containing 0.1 mM CC and HQ at different scan rates (from inner to outer: 0.02, 0.05, 0.08, 0.11, 0.14, 0.17, and 0.20 V∙s^−1^, respectively) (**A**). The linear relationships of the peak currents (I_p_) and the square root of the scan rates (*v*^1/2^) of HQ (**B**) and CC (**C**). The linear relationships of the peak potential (E_p_) and the natural logarithm of the scan rate (ln*v*) for HQ (**D**) and CC (**E**).

To analyze the electrochemical reaction kinetics of HQ and CC on the surface of the ERGO/MWCNTPE, the influence of the scan rates on the responses of HQ and CC was studied under optimized conditions using CV (Figure 6A). The oxidation peak currents (I_pa_) and reduction peak currents (I_pc_) of HQ and CC showed good linear relationships with the square root of the scan rate (*v*^1/2^). The relationships are as follows: I_pa, HQ_ (μA) = −14.92 *v*^1/2^ + 1.01 (R^2^ = 0.99), I_pc, HQ_ (μA) = 10.28 *v*^1/2^ − 0.48 (R^2^ = 0.99) for HQ (Figure 6B), and I_pa, CC_ (μA) = −11.7 *v*^1/2^ + 0.96 (R^2^ = 0.99), I_pc, CC_ (μA) = 17.17 *v*^1/2^ − 1.59 (R^2^ = 0.99) for CC (Figure 6C). The above results indicate a diffusion-controlled process on the surface of the modified electrode [48]. As shown in Figure 6D,E, the electrochemical reactions of HQ and CC on the sensing interface are quasi-reversible. The linear relationships between E_p_ and the natural logarithm of the scan rate (ln*v*) are E_pa, HQ_ (V) = 0.015 ln*v* (V∙s^−1^) + 0.13 (R^2^ = 0.99), E_pc, HQ_ (V) = −0.008 ln*v* (V∙s^−1^) + 0.03 (R^2^ = 0.99) for HQ, and E_pa, CC_ (V) = 0.012 ln*v* (V∙s^−1^) + 0.24 (R^2^ = 0.99), E_pc, CC_ (V) = −0.010 ln*v* (V∙s^−1^) + 0.14 (R^2^ = 0.99) for CC.

According to Nicholson’s theory [49], for quasi-reversible electrochemical reaction processes controlled by diffusion, the redox peak potential is linearly related to the natural logarithm of the scan rate by the following equation:

(1) $$E_{pa}=E^{\theta }+\frac{RT}{\alpha nF}\left[0.781+\ln\left(\frac{D^{1/2}}{k^{\theta }}\right)+{\ln\left(\frac{\alpha nFv}{RT}\right)}^{1/2}\right]$$

(2) $$E_{pc}=E^{\theta }-\frac{RT}{\left(1-\alpha \right)nF}\left[0.781+\ln\left(\frac{D^{1/2}}{k^{\theta }}\right)+{\ln\left(\frac{\left(1-\alpha \right)nFv}{RT}\right)}^{1/2}\right]$$

where $E^{\theta }$ is the formal potential, α is the electron transfer coefficient, $k^{\theta }$ is the standard rate constant, n is the electron transfer number, F is the Faraday constant, D is the diffusion coefficient, and T is the absolute temperature.

**Figure 7:**
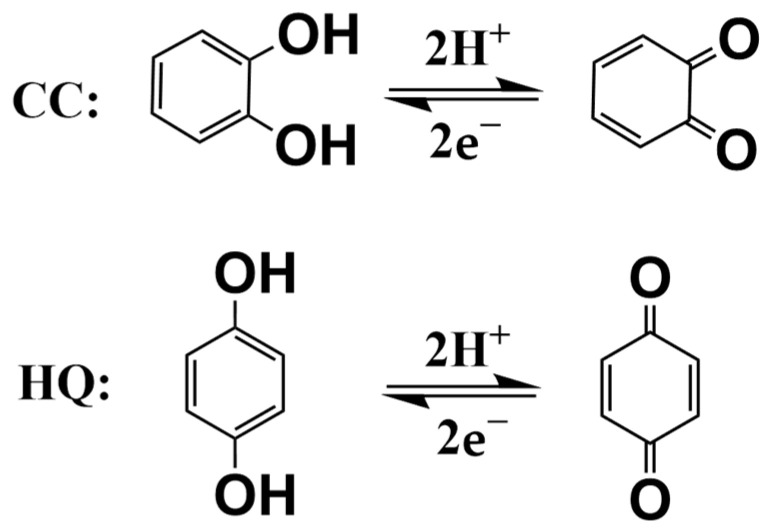
The electrochemical reaction mechanism of CC and HQ on the ERGO/MWCNTPE.

We calculated the transfer number of electrons (*n*) for HQ and CC, which were 2.4 (≈2) and 2.3 (≈2), and the α values, which were 0.358 and 0.454, respectively. Based on the conclusion in Section 2.5, the possible reaction mechanism of CC and HQ on the ERGO/MWCNTPE is shown in Figure 7, which is consistent with results reported in the literature [50].

### 2.7. Adsorption Quantity of CC on Different Electrodes

Chronocoulometry is an electrochemical technique in which a constant potential is set. It can be used to determine the adsorbed number of active species in a solution of freely diffusing active species. The electrochemical adsorption behavior of CC and HQ on both the bare MWCNTPE and the ERGO/MWCNTPE was investigated using chronocoulometry (Figure S3A,C). As shown in Figure S3B,D, the linear regression equations of the charge Q and t^1/2^ were Q_MWCNTPE_ (μC) = 1.03 t^1/2^(s^1/2^) − 1.16 (R^2^ = 0.99), Q_ERGO/MWCNTPE_ (μC) = 125.95 t^1/2^(s^1/2^) − 151.66 (R^2^ = 0.99) for CC, and Q_MWCNTPE_ (μC) = 1.04 t^1/2^(s^1/2^) − 1.18 (R^2^ = 0.99), Q_ERGO/MWCNTPE_ (μC) = 125.47 t^1/2^(s^1/2^) − 147.96 (R^2^ = 0.99) for HQ.

We combined these results with the Anson equation [51]:

(3) $$Q=2ncFAD_{0}^{1/2}t^{1/2}\pi^{-1/2}+Q_{dl}+nFA\Gamma_{0}$$

where n is the electron transfer number, c is the concentration of HQ or CC, F is the Faraday constant, $D_{0}$ is the diffusion coefficient of HQ or CC, $nFA\Gamma_{0}$ is the Faraday charge induced by the surface adsorbent, $Q_{dl}$ is the charge of the double electric layer, and A is the electrode surface area. Based on the results shown in Figure S4 [52,53], the effective areas (A) of the bare MWCNTPE and the ERGO/MWCNTPE were calculated to be 5.17 × 10^−3^ cm^2^ and 9.876 × 10^−3^ cm^2^, respectively. Using Equation (3), the saturated adsorption capacity ($\Gamma_{0}$) for HQ and CC on the bare MWCNTPE and the ERGO/MWCNTPE were calculated as follows: 1.18 × 10^−9^ mol∙cm^−2^, 7.76 × 10^−8^ mol∙cm^−2^ for HQ, and 1.16 × 10^−9^ mol∙cm^−2^, 7.76 × 10^−8^ mol∙cm^−2^ for CC. The adsorption quantity of HQ and CC on the ERGO/MWCNTPE was approximately 80 times greater than that on the bare MWCNTPE, indicating a significant contribution of ERGO to the enrichment of HQ and CC.

### 2.8. Individual and Simultaneous Determination of CC and HQ

**Figure 8:**
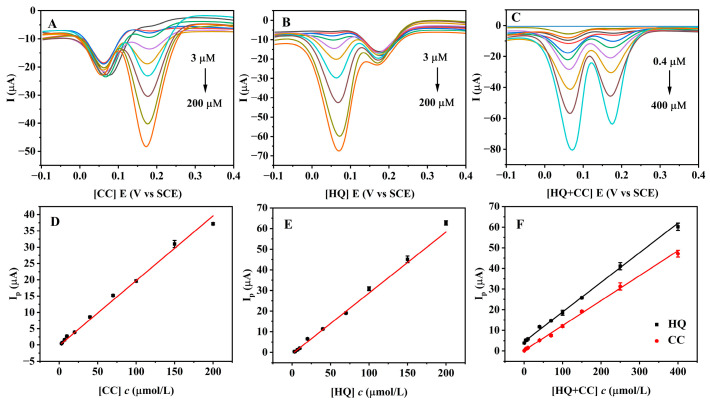
Differential pulse voltammograms of the ERGO/MWCNTPE in 0.2 M PBS (pH = 6.0) containing 50 μM HQ and different concentrations of CC (from inner to outer: 3, 4, 7, 10, 20, 40, 70, 100, 150 and 200 μM) (**A**), 50 μM CC and different concentrations of HQ (from inner to outer: 3, 4, 7, 10, 20, 40, 70, 100, 150 and 200 μM) (**B**), and CC and HQ of different concentrations (from inner to outer: 0.4, 4, 7, 10, 40, 70, 100, 150, 250 and 400 μM) (**C**). The corresponding linear relationships of the ERGO/MWCNTPE in 0.2 M PBS (pH = 6.0) containing 50 μM HQ and different concentrations of CC (3–200 μM) (**D**), 50 μM CC and different concentrations of HQ (3–200 μM) (**E**), and different concentrations of CC and HQ (0.4–400 μM) (**F**).

Differential pulse voltammetry (DPV) is an electrochemical technique with high sensitivity and resolution for detecting low concentrations of compounds. Therefore, the linear range, selectivity, and detection limits of the sensor were investigated through multiple DPV measurements in a 0.2 M PBS solution (pH = 6.0) under optimal experimental conditions. As shown in Figure 8A, with the response of HQ fixed at 50 μM and CC varying from 3 μM to 200 μM, the calibrated linear regression equation for CC was I_p_(μA) = 0.20 c (μM) − 0.13 (R^2^ = 0.99) (Figure 8D). Figure 8B shows that with the response of CC fixed at 50 μM and HQ varying from 3 μM to 200 μM, the calibrated linear regression equation for HQ was I_p_ (μA) = 0.29 c (μM) − 0.74 (R^2^ = 0.99) (Figure 8E). This indicates that selective determination of one analyte in the presence of the other is feasible.

The feasibility of the simultaneous determination of CC and HQ using this modified electrode was evaluated in multiple ways. The response obtained with DPV when the concentrations of CC and HQ were varied simultaneously is shown in Figure 8C. The calibrated linear regression equations for CC and HQ were I_p, CC_ (μA) = 0.12 c (μM) + 0.24 (R^2^ = 0.99) (0.4–400 μM) and I_p, HQ_ (μA) = 0.14 c (μM) + 4.56 (R^2^ = 0.99) (0.4–400 μM), respectively (Figure 8F). The detection limits of CC and HQ were 0.083 μM and 0.028 μM (S/N = 3), respectively. This indicates that the ERGO/MWCNTPE can be used for the separation and simultaneous determination of CC and HQ. As shown in Table 1, compared with other reported electrochemical sensors for CC and HQ, our sensor not only offers the advantage of easy preparation but also has low detection limits.

### 2.9. Reproducibility, Repeatability, and Selectivity

**Figure 9:**
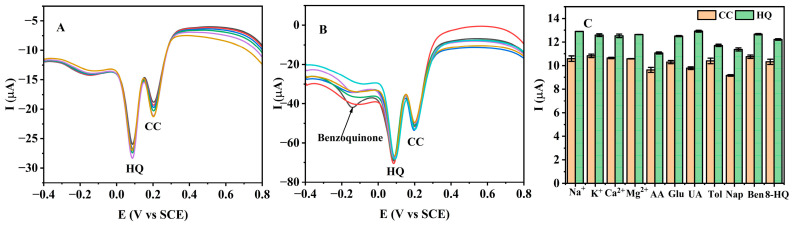
Electrochemical response of 0.1 mM CC and HQ on a single ERGO/MWCNTPE for six parallel measurements (**A**). Electrochemical response of 0.1 mM CC and HQ on six different ERGO/MWCNTPEs (**B**). Evaluation of the selectivity of the sensor (**C**).

The reproducibility and repeatability of the sensor were examined using SWV. The relative standard deviations (RSDs) were 5.65% and 4.73% for one ERGO/MWCNTPE used for the parallel determination of 0.1 mM HQ and CC (Figure 9A) over six measurements. For six different ERGO/MWCNTPEs used for the parallel measurement of 0.1 mM HQ and CC (Figure 9B), the RSDs were 5.5% and 1.18%. The literature studies suggest that the peak around −0.17 V is likely due to benzoquinone. These results indicate the good reproducibility and repeatability of the sensor.

In addition, the selectivity measurements showed no interference for 0.1 mM HQ and CC (containing 0.3 mM Na^+^) by 0.1 mM benzene (Ben) and naphthalene (Nap), 0.5 mM glucose (Glu), ascorbic acid (AA), toluene (Tol), uric acid (UA), 8-hydroxyquinoline (8-HQ) or 10 mM Ca^2+^, Mg^2+^, and K^+^, with RSDs of less than 5% (Figure 9C). The good selectivity may be due to the fact that HQ and CC are small polar molecules that can strongly interact with the oxygen functional groups (such as hydroxyl and carboxyl) and defect structures of ERGO through mechanisms involving hydrogen bonds, π-π interactions and adsorption, in addition to the good peak separation of ERGO.

### 2.10. Real Sample Analysis

In order to verify the practicality of the sensor, the CC and HQ contents in the tap water sample and urine sample were determined using the standard addition method with DPV. The results are shown in Table 2. The sensors demonstrated good recoveries for each sample. The average recoveries of HQ and CC were 98.9% and 100.5% for the tap water sample, and 100.1% and 100.6% for the urine sample, respectively, and the RSDs were both lower than 5.0%, indicating the sensor’s high accuracy and its potential for preventing water pollution and human health monitoring.

## 3. Materials and Methods

### 3.1. Reagents and Samples

Graphene oxide (GO), sodium dihydrogen phosphate dihydrate (NaH_2_PO_4_·2H_2_O), disodium hydrogen phosphate dodecahydrate (Na_2_HPO_4_·12H_2_O), glucose (Glu) and ascorbic acid (AA) were purchased from Shanghai Yuanye Bio-Technology Co., Ltd. (Shanghai, China). Multi-walled carbon nanotube (MWCNT, Ø = 10–20 nm purity ≥ 95 wt%) powder was bought from Suzhou Carbonfund Graphene Technology Co., Ltd. (Suzhou, China). Catechol (CC) and uric acid (UA) were purchased from Aladdin Reagent Co., Ltd. (Shanghai, China). Hydroquinone (HQ), K_3_[Fe(CN)_6_], K_4_[Fe(CN)_6_], KCl, NaCl, CuCl_2_, MgCl_2_, graphite, benzene (Ben), naphthalene (Nap), toluene (Tol) and 8-hydroxyquinoline (8-HQ) were bought from Sinopharm Chemical Reagent Co., Ltd. (Shanghai, China). Liquid paraffin was purchased from Jiangsu Qiangsheng Functional Chemical Co., Ltd. (Changshu, China). Copper wire was purchased from the local supermarket. All reagents were of analytical grade and used without further purification. All solutions were prepared with ultra-pure water. The tap water sample was prepared by injecting a quantity of tap water into a small beaker and diluting it to 5 mL with 0.2 M PBS buffer (pH = 6). The tap water was collected from our laboratory. The urine sample was taken from a healthy volunteer when it was ethically permissible to do so and was diluted to the desired concentration with 0.2 M PBS buffer (pH = 6) as the supporting electrolyte.

### 3.2. Apparatus

Scanning electron microscope (SEM) of the WMCNTs and ERGO was carried out with an FEI-Sirion200 (FEI, Hillsboro, OR, USA). X-ray photoelectron spectroscopy (XPS) measurements were carried out on an ESCALAB Xi^+^ X-ray photoelectron spectrometer (Thermo Fisher Scientific, Waltham, MA, USA). Raman spectroscopy was carried out by the inVia Qontor (Renishaw PLC, Wotton-under-Edge, UK) (532 nm and 0.5 laser power). Electrochemical impedance spectroscopy (EIS) was carried out with a Zennium electrochemical workstation (Zahner, Kronach, German). Electrochemical measurements were carried out with a CHI 830D electrochemical workstation (Chenhua Instrument Co., Ltd., Shanghai, China). The conversion of chemical data into electrical signals was facilitated by CHI830D (22.04) software and the data were calibrated and processed using Origin 2022 (9.90.225) software. A typical three-electrode cell with a 5 mL volume was used for all electrochemical experiments, with a bare or modified electrode as the working electrode, a platinum wire as the counter electrode, and a saturated calomel electrode (SCE) as the reference electrode. The analytical techniques used were cyclic voltammetry (CV), square wave voltammetry (SWV), differential pulse voltammetry (DPV), and electrochemical impedance spectroscopy (EIS).

### 3.3. Preparation of Carbon Paste Microelectrodes

#### 3.3.1. Fabrication of Bare MWCNTPEs

Bare MWCNTPEs were fabricated according to methods listed in the literature [63]. First, homemade microelectrodes were fabricated by inserting a copper wire (Φ = 500 μm) into a pipette with a tip diameter of 100 μm, burning and melting the tail end of the pipette by using an alcohol lamp, and followed by natural cooling to fix the copper wire. Thereafter, MWCNT paste was obtained by mixing 5 mg MWCNT powder with 15 μL of liquid paraffin and grinding the mixture by hand in a small agate mortar until a homogeneous paste was obtained. Lastly, a quantity of MWCNT paste was packed into the homemade microelectrode, and the electrode surface was polished manually on a piece of weighting paper until it was smooth and shiny.

To renew the surface of the carbon paste microelectrodes, they were first sonicated in ethanol for 3 min to remove the old carbon paste, and then the new paste was packed into the microelectrodes.

#### 3.3.2. Fabrication of the ERGO/MWCNTPE

First, 2 mg of GO was dispersed in 1 mL of ultra-pure water and sonicated for 3 h. Afterward, the denoted GO solution was obtained by mixing the dispersed GO solution with 0.1 M phosphate-buffer solution (pH = 7.4) in a 1:1 ratio. To fabricate the ERGO/MWCNTPEs, the MWCNTPE was placed in 5 mL of a 1 mg∙mL^−1^ GO solution. Cyclic voltammetry was performed for 10 cycles at a scan rate of 100 mV∙s^−1^ in the range of 0 to −1.7 V (vs. SCE), followed by drying at room temperature.

### 3.4. Statistical Analysis

The peak currents of three replications of all experiments are expressed as the mean ± SD. Statistical analysis was performed using Origin 2022 (9.90.225) (OriginLab Corporation, Northampton, MA, USA), with the statistical significance level set to α < 0.05. Linear baseline correction was applied to address the peak shoulder overlap of CC and HQ resulting from molecular competition. Real sample analysis was performed on tap water and urine samples using the standard addition method to assess the accuracy of the developed sensors.

## 4. Conclusions

A novel electrochemical sensor for the simultaneous detection of CC and HQ was achieved in this work based on an electrochemically reduced graphene oxide (ERGO)-modified disposable MWCNT carbon paste microelectrode using one-step electrodeposition. The ERGO-MWCNT exhibits a high specific surface area, catalytic activity, and electrical conductivity. The sensing interface was characterized, the sensing mechanism was discussed, and the performance of the sensor was evaluated. The sensor offers advantages such as simple operation, low cost, and good performance, including high sensitivity, selectivity, and accuracy. It has promising applications in environmental protection and human health monitoring. Moreover, the use of a disposable carbon paste electrode and ERGO in the construction of an electrochemical sensor for the simultaneous detection of CC and HQ may provide a new approach for developing electrochemical sensors for environmental estrogens and benzene metabolite phenol in humans.

## Figures and Tables

**Table 1:** Analytical performance comparison of the ERGO/MWCNTPE for CC and HQ detection with other reported electrodes.

Electrodes	Linear Range (μM)		Detection Limit (μM)		Ref.
	CC	HQ	CC	HQ	
COFs/MWCNT/GCE	4–450	4–450	0.36	0.38	[54]
AuNPs/Fe_3_O_4_-GO/GCE	2–145	3–137	0.8	1.1	[55]
aGO/SCPE	1–350	1–312	0.182	0.27	[56]
CNT-SH@Au-GR/GCE	11–126	55–125	1.0	4.2	[57]
PFHSAAC/GCE	1–150	1–200	0.423	0.357	[58]
Ce-MOF/CNTs/GCE	5–50	10–100	3.5	5.3	[5]
Pt/C_60_/PGE	50–1500	50–1100	2.97	2.19	[59]
CuO-CNF/GCE	0–150	3–80	2.0	1.0	[60]
PEDOT/GO/GCE	2–400	2.5–200	1.6	1.6	[61]
Co_3_O_4_@carbon/GCE	0.6–116.4	0.8–127.1	0.03	0.03	[62]
ERGO/MWCNTPE	0.4–400	0.4–400	0.083	0.028	This work

COFs: Covalent organic frameworks; AuNPs: gold nanoparticles; aGO: activated graphene oxide; SCPE: screen-printed carbon electrode; CNT-SH@Au-GR: gold nanoparticle–graphene nanohybrid-bridged 3-amino-5-mercapto-1,2,4-triazole-functionalized multi-wall carbon nanotubes; PFHSAAC: perilla frutescens; MOF: metal–organic frameworks; CNTs: carbon nanotubes; PGE: pyrolytic graphite electrode; CNF: carbon nano-fragments; PEDOT: poly(3,4-ethylenedioxythiophene).

**Table 2:** Application of the sensor in the detection of CC and HQ in tap water sample and urine sample (n = 3).

Sample	Added (μM)		Found (μM)		Recovery (%)		RSD (%)	
	HQ	CC	HQ	CC	HQ	CC	HQ	CC
Tap water	50	50	48.96	50.50	97.92	101.00	4.86	1.89
	100	100	99.28	100.67	99.28	100.67	1.18	0.98
	150	150	149.14	149.67	99.42	99.78	4.59	4.05
Urine	50	50	49.47	50.81	98.94	101.62	1.61	5.55
	100	100	100.78	99.58	100.78	99.58	2.55	3.88
	150	150	150.71	150.83	100.47	100.55	0.63	3.09

## Data Availability

Data are contained within the article or Supplementary Material.

## Supplementary Materials

The following supporting information can be downloaded at https://www.mdpi.com/article/10.3390/ijms25189829/s1.
