# Supplementary material for: Simultaneous Electrochemical Detection of Catechol and Hydroquinone Based on a Carbon Nanotube Paste Electrode Modified with Electro-Reduced Graphene Oxide
Source: Int J Mol Sci. 2024 Sep 11;25(18):9829. doi: 10.3390/ijms25189829 (PMC11432359; doi:10.3390/ijms25189829)
Supplement: Supplementary file 1 [file ijms-25-09829-s001.zip › ijms-3176771-supplementary.pdf]

Electronic Supplementary Material on the *International Journal of Molecular Sciences* publication entitled

Simultaneous Electrochemical Detection of Catechol and Hydroquinone Based on a Carbon Nanotube Paste Electrode Modified with Electro-Reduced Graphene Oxide

Tingfei Chen[1,†], Chao Liu[1,2,3,†], Xiaojun Liu[1,2,3], Chunnan Zhu[1,2,3], Dongyun Zheng[1,2,3,*]

1. School of Biomedical Engineering, South-Central Minzu University, Wuhan 430074, China;

2. Key Laboratory of Cognitive Science, State Ethnic Affairs Commission, Wuhan 430074, China;

3. Hubei Key Laboratory of Medical Information Analysis and Tumor Diagnosis & Treatment, Wuhan 430074, China

* Correspondence: wintercloud@mail.scuec.edu.cn

† These authors contributed equally to this work.

Optimization of experimental conditions

To enhance the sensitivity of CC detection, the sensor's fabrication and operational parameters were systematically optimized. This optimization process involved adjusting the concentration of graphene oxide (GO), the number of electrochemical reduction cycles, the enrichment potential, the enrichment time, and the concentration of phosphate-buffered saline (PBS) buffer, utilizing square wave voltammetry (SWV) as the analytical technique. The outcomes of these optimizations are illustrated in Figure S1. The controlled variable method was applied throughout, with the concentration of CC maintained at 0.1 mM.

The influence of the ERGO layer is illustrated in Figures S1(A) and S1(B). Increasing the GO concentration while keeping the scan cycles constant resulted in a rise in the oxidation current of CC, with a peak at 1 mg·mL^{-1}. Similarly, increasing the number of scan cycles while maintaining the GO concentration led to a peak at 10 cycles. This finding indicates that electrodeposition with 1 mg·mL^{-1} GO for 10 cycles maximizes the ERGO surface area, providing the greatest number of active sites and enhancing electron transfer. However, a thicker ERGO layer hinders electron transfer, reducing the oxidation peak current of CC. Thus, the optimal electrodeposition conditions are 10 cycles of CV scanning with 1 mg·mL^{-1} GO.

The influence of accumulation potential is shown in Figure S1(C). The peak current gradually increased from −0.6 to −0.2 V and then decreased after peaking at −0.2 V, indicating that the most sensitive response occurred at −0.2 V. Figure S1(D) illustrates that the CC peak current increased rapidly as the accumulation time increased from 20 to 50 seconds and then decreased after 50 seconds, reaching a maximum at 50 seconds. Therefore, −0.2 V and 50 seconds were selected as the optimal accumulation potential and time for the determination of CC.

The influence of PBS concentration is shown in Figure S1(E). As the concentration of the substrate increases from 0.1 M to 0.35 M, the current reaches its maximum at 0.2 M, further demonstrating that the substrate concentration facilitates the diffusion of CC on the electrode surface. Therefore, the optimal PBS concentration is 0.2 M.

To optimize the detection conditions, the pH value of the supporting electrolyte was adjusted, as it significantly affects the electrochemical response of the analyte. A series of 0.2 M phosphate-buffered solutions with varying pH values was prepared. The influence of pH on the response current of CC and HQ is illustrated in Figure S2. It is evident that the optimal response is achieved at pH 6.0, indicating that this pH enhances the interaction between ERGO-MWCNTs and the analytes CC and HQ.

Calculation of the effective area of the electrode

To analyze the effective area of the bare MWCNTPE and the ERGO/MWCNTPE, the electrochemical responses of different electrodes were investigated in 5 mM $[Fe(CN)_6]^{3-/4-}$ solution containing 1 M KCl at various scan rates using cyclic voltammetry (CV). As shown in Figure S4(A) and Figure S4(C), reversible redox peaks of the probe were observed for both the bare MWCNTPE and the ERGO/MWCNTPE. Furthermore, as indicated in Figure S4(B) and Figure S4(D), the peak current of the probe increased linearly with the square root of the scan rate as follows: $I_{pa,\ MWCNTPE}$ (μA) = 20.00 $v^{1/2}$ + 0.895 (R^2 = 0.999) and $I_{pa,\ ERGO/MWCNTPE}$ (μA) = 36.69 $v^{1/2}$ + 0.003 (R^2 = 0.999), respectively, according to the Randles–Sevcik equation [1].

$$\text{When } 25\ ℃,\ i_p = (2.69 \times 10^5) \times n^{3/2} v^{1/2} D^{1/2} AC \quad \text{(S1)}$$

A: electrode area (cm^2), D: diffusion coefficient (5 mM $K_3[Fe(CN)_6]/K_4[Fe(CN)_6]$ + 1 M KCl, D_{ox}=7.63×10^{-6} $cm^2{\cdot}s^{-1}$) [2], C: solution concentration ($mol{\cdot}cm^{-3}$), v: scanning rate ($V{\cdot}s^{-1}$), and i_p: peak current (A). Combining Equation (S1) with the results from Figure S4(B) and Figure S4(D), the effective areas of the bare MWCNTPE and the ERGO/MWCNTPE were 5.17 × 10^{-3} cm^2 and 9.88 × 10^{-3} cm^2, respectively. This finding confirms that modification with ERGO significantly increased the electrochemically active area of the electrode, thereby enhancing the adsorption capacity of the ERGO/MWCNTPE for CC and HQ.

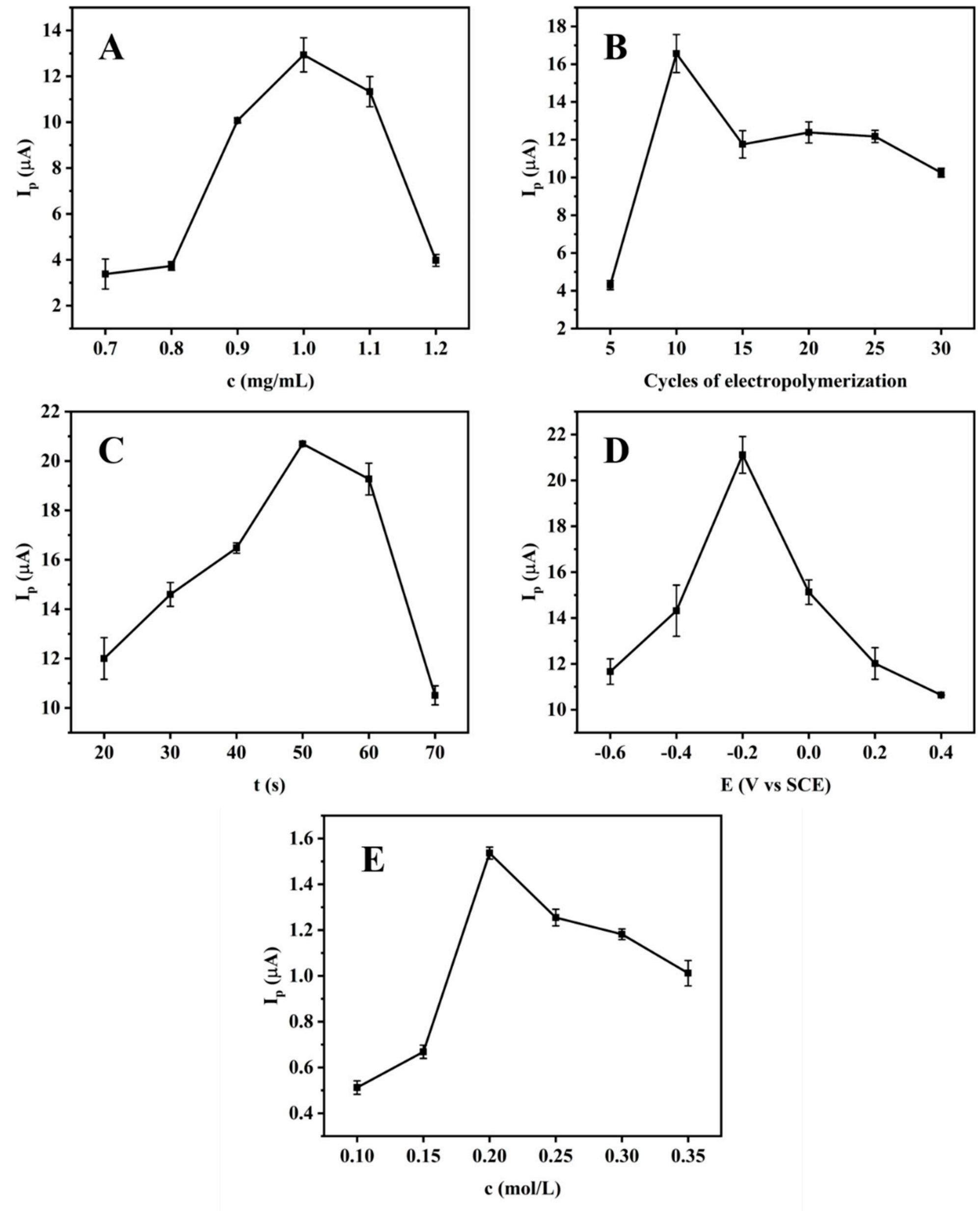

Figure S1. Influence of the concentration of GO (A), the number of electrochemical reduction cycles (B), the enrichment time (C), the enrichment potential (D) and the concentration of PBS (E).

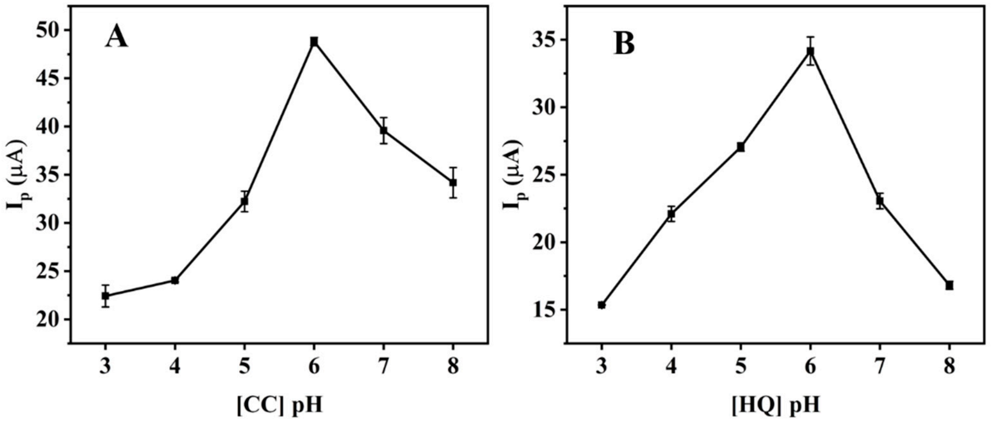

Figure S2. Influence of the pH values of the electrolyte on the electrochemical response of CC (A) and HQ (B).

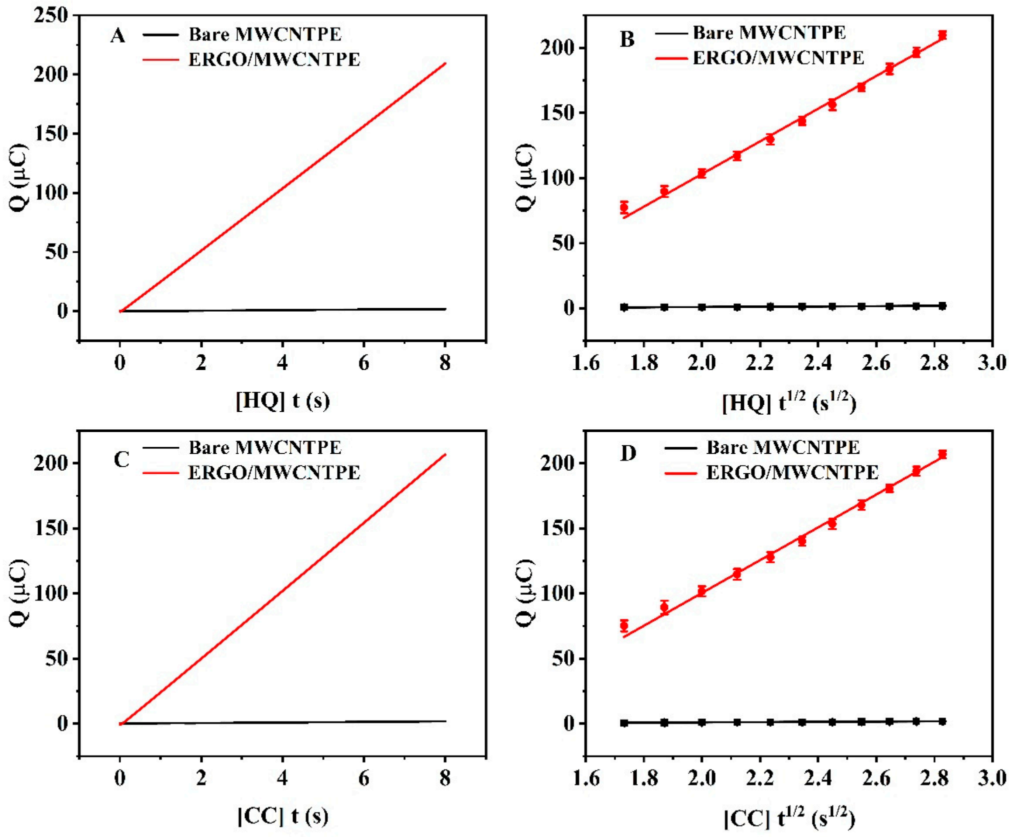

Figure S3. Chronocoulometric curves of the bare MWCNTPE and the ERGO/MWCNTPE in 0.2 M PBS (pH = 6.0) containing 0.1 mM HQ (A) and CC (C). The linear relationship between Q and $t^{1/2}$ for HQ (B) and CC (D).

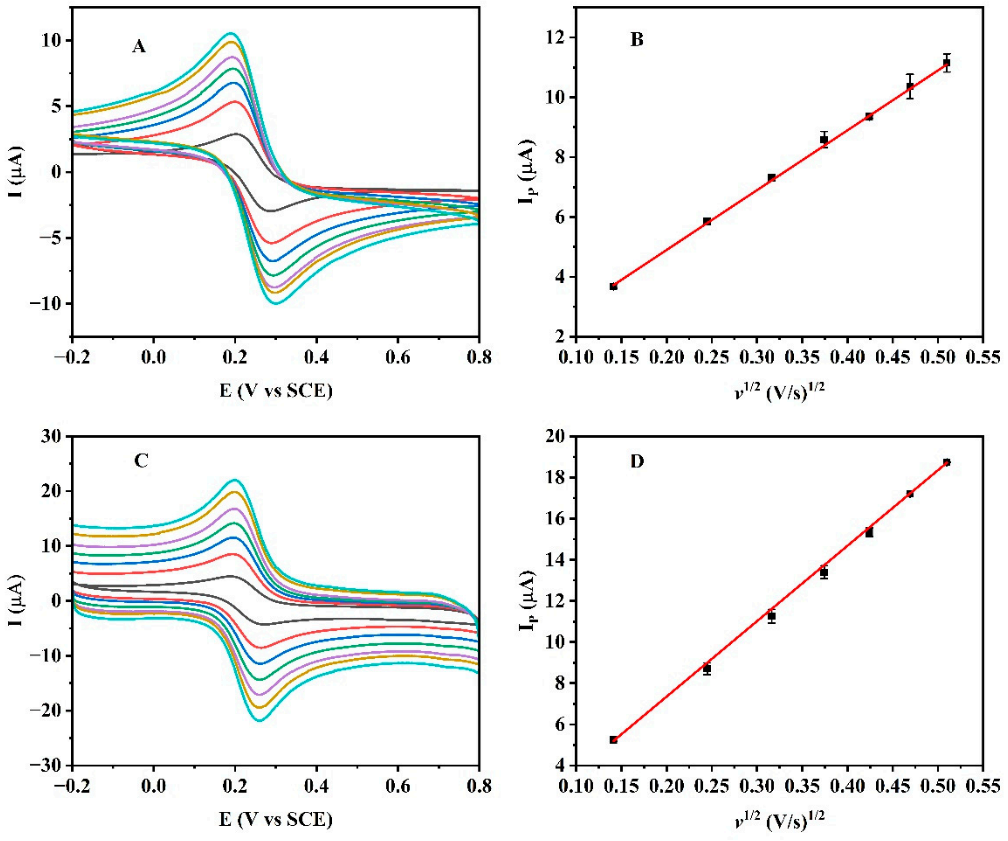

Figure S4. Cyclic voltammograms of I_p versus $v^{1/2}$ for the bare MWCNTPE (A) and the ERGO/MWCNTPE (C) in a 5 mM $[Fe(CN)_6]^{3-/4-}$ solution containing 1 M KCl at different scan rates (from inner to outer: 0.02, 0.06, 0.1, 0.14, 0.18, 0.22 and 0.24 $V \cdot s^{-1}$), and the linear relationship of I_p versus $v^{1/2}$ on the bare MWCNTPE (B) and the ERGO/MWCNTPE (D).

References

1. Ngamchuea, K.; Eloul, S.; Tschulik, K.; Compton, R.G. Planar diffusion to macro disc electrodes—what electrode size is required for the Cottrell and Randles-Sevcik equations to apply quantitatively? *J. Solid State Electrochem.* **2014**, *18*, 3251-3257, doi:http://doi.org/10.1007/s10008-014-2664-z.
2. Sundfors, F.; Bobacka, J.; Ivaska, A.; Lewenstam, A. Kinetics of electron transfer between $[Fe(CN)_6]^{3-/4-}$ and poly(3,4-ethylenedioxythiophene) studied by electrochemical impedance spectroscopy. *Electrochim. Acta* **2002**, *47*, 2245-2251, doi:http://doi.org/10.1016/S0013-4686(02)00063-4.
